# Effects of Spacing on Sentence Reading in Chinese

**DOI:** 10.3389/fpsyg.2021.765335

**Published:** 2021-11-10

**Authors:** Gaisha Oralova, Victor Kuperman

**Affiliations:** Department of Linguistics and Languages, McMaster University, Hamilton, ON, Canada

**Keywords:** reading, Chinese, eye movements, inter-word spacing, segmentation probability

## Abstract

Given that Chinese writing conventions lack inter-word spacing, understanding whether and how readers of Chinese segment regular unspaced Chinese writing into words is an important question for theories of reading. This study examined the processing outcomes of introducing spaces to written Chinese sentences in varying positions based on native speaker consensus. The measure of consensus for every character transition in our stimuli sentences was the percent of raters who placed a word boundary in that position. The eye movements of native readers of Chinese were recorded while they silently read original unspaced sentences and their experimentally manipulated counterparts for comprehension. We introduced two types of spaced sentences: one with spaces inserted at every probable word boundary (heavily spaced), and another with spaces placed only at highly probable word boundaries (lightly spaced). Linear mixed-effects regression models showed that heavily spaced sentences took identical time to read as unspaced ones despite the shortened fixation times on individual words (Experiment 1). On the other hand, reading times for lightly spaced sentences and words were shorter than those for unspaced ones (Experiment 2). Thus, spaces proved to be advantageous but only when introduced at highly probable word boundaries. We discuss methodological and theoretical implications of these findings.

## Introduction

One of the differences between Chinese and many other languages is that the Chinese writing system does not have inter-word spacing, thus offering no overt visual cues for identifying word boundaries. This fact gave rise to a large-scale ongoing inquiry into how Chinese readers segment print information into chunks for processing and what guides this segmentation process. The present paper contributes to this inquiry by studying the effects of introducing space symbols at specific character transitions on word and sentence recognition. We begin with a brief review of the relevant literature and orthographic system of written Chinese and follow up with an outline of the present study.

The central unit of the Chinese writing system is a box-like character which normally corresponds to a monosyllabic morpheme. A single word can consist of one or several characters (or *zi*). Similarly, one character, or *zi*, can constitute a single word or can be part of another multi-character word. A Chinese word, or *ci*, as defined by the traditional grammar, is a linguistic unit which denotes a meaning and a pronunciation, may stand alone to constitute a sentence and can be a grammatical unit on its own (Hoosain, 1991, 1992).

Chinese readers sometimes disagree on what constitutes a word or where a word's boundaries are in a given sentence. This observation is the central finding of a study by Liu et al. (2013) in which they asked Chinese readers to identify word boundaries by inserting slashes between words in a natural unspaced text. Liu et al. (2013) found that judgments about where the boundaries should be placed varied widely across participants. The average inter-rater agreement on segmentation judgments was 64%. Liu et al. (2013) also observed that Chinese raters tended to group characters into larger informational units and that their segmentation was influenced by syntactic categories. For example, they combined consecutive nouns to form a single chunk and combined function words with content words to form a single unit.

In an earlier study, Hoosain (1992) instructed Chinese speakers to segment sentences into words and also found that they had a substantial degree of disagreement on what constitutes a word boundary. Interestingly, when asked to explain their word boundary decisions, participants indicated that they aimed to separate “one thing” or “one idea” with boundaries. Hoosain (1992) explains that reading for meaning is a cause of divergent segmentation decisions, because units of meaning may go beyond character and word units. Ultimately, what a reader considers “one thing” or “one idea” could vary depending on their focus at the time of processing. Despite the abundance of evidence on word boundary disagreement, other research shows that certain word properties influence a range of reading measures in Chinese, signifying that words are psychologically real in Chinese minds (see Li et al., 2014).

### Effects of Spacing on Reading Behavior

In order to better understand what constitutes a unit of processing in Chinese and other languages without overt segmentation cues, researchers have often introduced spaces into a normally unspaced text in experimental studies of Chinese word segmentation. This manipulation examines if spaces benefit readers of unspaced languages by facilitating the segmentation process and, importantly, increase reading speed or improve comprehension. The present study makes use of this manipulation as well.

The role of spacing is well-documented in alphabetic languages with conventional inter-word spaces. For instance, when inter-word spacing is eliminated, the reading rate of English readers is slowed down by 30–50% (Rayner and Pollatsek, 1996). This is because spacing guides saccadic movements of the eye and helps word recognition in general (Pollatsek et al., 1990; Epelboim et al., 1994). Nevertheless, this facilitatory effect of spacing is not universal across all languages, and certainly not in the languages that do not use spaces. In an eye-tracking study where Japanese speakers read spaced and unspaced texts in pure Hiragana and mixed Kanji-Hiragana scripts, Sainio et al. (2007) found that spaces did not facilitate text reading rate (measured in words per minute) either in the syllabic Hiragana script nor in the mixed script condition. Facilitatory effects were found only at the word-level analysis (word fixation duration measures) and only for the mixed Kanji-Hiragana script. The proposed explanation for facilitation was that in Japanese, characters frequently appear at the beginning of words, and as a result, in the mixed Kanji-Hiragana script, their occurrence serves as a segmentation cue for word boundaries (Sainio et al., 2007). In a study with another non-spaced language, Winskel et al. (2009) tested English-Thai bilinguals when they were presented with spaced and non-spaced Thai texts and found that sentence reading times were 5% longer in the spaced condition than the non-spaced condition. The authors suggest the lack of facilitatory effects from spacing in Thai was due to the visual salience of words in the segmented text. This resulted in the words attracting more fixations, which led to an increase in sentence reading times. More importantly, Thai has certain language-specific word segmentation cues, such as letter clusters (vowels occurring before the consonants at syllable beginnings, e.g., 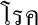 written as /o:rk/ *disease*) or tone markers (placed above syllables or lexemes, e.g., 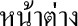 /na:2ta:η1/ *window*), which is redundant with additional segmentation information in the form of spacing. Thus, in both Japanese and Thai, there are other visual characteristics of the printed text serving as word boundary cues that affect segmentation decisions. In these circumstances, the addition of spaces brings about null or inhibitory effects.

Similar inhibitory effects of spacing were found in some studies on Chinese reading. Bai et al. (2008) investigated whether the introduction of spaces into naturally unspaced Chinese helps reading. They used four types of sentences in their spacing conditions: (i) unspaced sentences, (ii) sentences where spaces were between words, (iii) sentences where spaces were placed in positions such that non-words were created, and (iv) sentences with spaces between each character. The researchers found that readers made shorter fixations on words in condition (ii), and the longest fixations on words in conditions (iii) and (iv). Although fixation times were shorter on words demarcated by spaces, these benefits were short-lived, and no differences were found in sentence reading times whether the sentences were fully unspaced or spaced just at the word level. Another eye-tracking study by Inhoff et al. (1997) presented Chinese sentences in three conditions: normal non-spaced, word-spaced with a space between every word, and non-word spaced where spaces were inserted such that character combinations formed non-words. Results did not show any differences between conditions, neither in total sentence reading times, nor in word fixation times. Interestingly, Bassetti (2009) compared sentence reading times and comprehension rates of native and non-native Chinese readers when they read texts with inter-word spacing and unspaced texts in Chinese. These results likewise did not indicate any signs of facilitated reading for Chinese texts with inter-word spacing in either of the groups.

On the contrary, some studies show beneficial effects of spaces in Chinese. For example, Hsu and Huang (2000a,b) found that although spacing between words did not facilitate reading, sentence reading time was reduced when a space was inserted to guide segmentation decisions in reading of overlapping ambiguous strings. Interestingly, some other studies also showed the beneficial effects of spacing, but they were observed only at the word-level, with sentence reading times being identical in spaced and unspaced conditions. For instance, Cui et al. (2014) hypothesized that spacing between words would allow for a more focused allocation of attention, which would enhance the parafoveal preview benefit compared to the control unspaced condition. In their study, using a gaze boundary paradigm with a correct and incorrect preview character, they showed that there was a bigger preview effect in the spaced condition, but only for one-character words. Cui et al. (2014) concluded that overt boundary cues enhance allocation of attention and lead to more efficient parafoveal processing in Chinese reading. In another study, Zang et al. (2013) examined children and adults' eye movement behavior when they read spaced and unspaced texts in Chinese. Zang et al. (2013) showed that inter-word spacing decreased first pass reading times (first fixation duration, single fixation duration and gaze duration) in both groups, indicating that inter-word spacing facilitates the word identification process. This word-level advantage in Cui et al. and Zang et al. runs counter to a logically possible hypothesis that introduction of spaces causes upcoming words to be located further away from the current fixation and thus might decrease the efficiency of parafoveal preview.^1^ However, sentence reading times in Zang et al.'s data were similar for spaced and unspaced conditions. They concluded that introducing spaces between words may help early segmentation, but the unusual visual presentation of the spaced text may cause a disruption to online global text comprehension. A trade-off between disruption and facilitation results in a statistically unreliable difference in total sentence reading times.

Cumulatively, the studies presented above indicated that, despite the short-lived advantage in reading speed at the word-level, by and large, spaces fail to significantly facilitate sentence reading times in Chinese, but do not appear to disrupt processing either.

### Statistical Cues During Word Segmentation

Earlier investigations of whether spaces inserted at character transitions help or hinder Chinese word segmentation have led to mixed results. Importantly, to our knowledge, not all of the studies mentioned above used a range of segmentation probabilities to guide the experimental decision of where to place spaces to demarcate word boundaries in Chinese texts. Consequently, it is possible that in previous studies spaces were put in places which some readers may have found counterintuitive. Yet, the statistical probabilities of character transitions either co-occurring within a word or straddling a word boundary are known to serve as efficient cues to reading in Chinese (see Inhoff and Wu, 2005, and discussion above). For instance, Zang et al. (2016) assessed whether Chinese readers segment words according to how likely a character was to appear as a single-character word or as a part of another two-character word. Results showed that the preview benefit from the second character was reduced when the first character was more likely to be a single character word. Zang et al. (2016) proposed that the first character acted like an “anchor” to signify that there is a word boundary, and hence, any additional characters to the right of fixation were not processed to the same degree prior to fixation. In another study, Yen et al. (2012) embedded two-character words in sentences and manipulated the contrast between the probabilities of the ending character (C2) of the target word (C12) being used as a word beginning or ending in all words containing it. They found that the probability of within-word positions affected character-to-word assignment and translated into longer reading times in lower-probability combinations of characters. In sum, Yen et al.'s (2012) and Zang et al.'s (2016) findings provide evidence that the segmentation probability of characters between and within words plays a crucial role in word segmentation and eye-movement control in Chinese.

We acknowledge that spacing is only one method of drawing readers' attention to segmentation cues, which interferes with the common visual layout of Chinese and may introduce artificial oculomotor and attentional demands on reading. Other artificial, less disruptive segmentation cues have been fruitfully used in the field, such as color grouping of words indicating a word boundary. Color marking of word boundaries consistently showed a beneficial effect on eye movement parameters (e.g., Perea and Wang, 2017; Zhou et al., 2018). We opted for the use of spacing for comparability of the present results with a broader existing literature in the field, and also for its practicality. If one of the manipulations of spacing were to lead to sizable consistent benefits in reading times at the word or sentence levels, spacing can be typographically implemented in Chinese texts for language learners or proficient readers with greater ease than, say, font coloring.

The literature above motivates the present study, which takes into account segmentation probabilities in an eye-tracking study of natural unspaced Chinese sentences and their spaced counterparts (see Zang et al., 2016). In the remainder of the Introduction, we introduce the critical experimental manipulation and predictions of our study.

### The Present Study

It is logical to assume that a segmentation cue like a space is the most beneficial when it is applied in an appropriate position in a sentence, for instance, at a transition between characters that is undoubtedly a word boundary. Conversely, inserting a space between characters that undoubtedly belong to the same word is likely disruptive to reading. Yet, all too often Chinese readers disagree on where the word boundaries are (Liu et al., 2013; Wang et al., 2015). That is, only a few character transitions are clearly fit or unfit for space insertion in Chinese. To our knowledge, no experimental study so far has exploited naturally occurring differences in segmentation probabilities to systematically examine the range of efficiency that spaces may offer as potential segmentation cues and the variable impact that such cues may have on word and sentence reading in Chinese.

We made use of segmentation judgments for word boundaries reported in Liu et al. (2013) and Wang et al. (2015) to create three experimental conditions based on their stimulus sentences: a natural unspaced condition; a heavily spaced condition, where spaces were inserted between a large number of character transitions (the transitions where at least 5% of raters agreed on a word boundary); and a lightly spaced condition, where spaces were inserted only in highly probable transitions. We defined a highly probable transition as a location where at least 90% of raters agreed to place a word boundary. All conditions used the same sentences and only differed in the amount of spacing. The rationale behind this setup was to explore whether insertion of spaces at transitions between characters in a sentence that varied in their suitability as word boundaries would have a detrimental or beneficial effect on reading times both in experimentally manipulated sentences and in natural unspaced sentences in written Chinese. The three conditions were distributed between two experiments conducted with two different groups of participants from the same participant pool. The first experiment included the heavily spaced and the unspaced conditions, whereas the second experiment included the lightly spaced and the unspaced conditions. The unspaced conditions were identical between the two experiments. We provide the motivation for our two experiments and their details in the Methods section below.

With a relatively large stimulus set (220 sentences), this study aims at exploring the existing uncertainty regarding the role of spacing in Chinese sentence reading and serves as a high-power extension of previous studies (see the literature review above). This study is novel in that it examines the role of probabilities of spaces as segmentation cues at a larger scale throughout entire sentences rather than in one or two specific positions in the sentence. The chosen experimental design enables us to examine the following question of interest. We ask whether spacing has an effect on Chinese reading of individual words and at the level of sentences in lightly and heavily spaced conditions. We expect to see longer sentence reading times for the heavily spaced condition compared to the unspaced one, as spaces at less probable word boundaries will be unexpected, and thus potentially disruptive for at least some readers. Additionally, adding spaces may either decrease parafoveal pre-processing efficiency and subsequently prolong reading times on individual words or helpfully guide the reader's attention to segmentation cues and thus shorten reading times (e.g., Cui et al., 2014). It is also possible that spaces at highly probable word boundaries (the lightly spaced condition) may facilitate segmentation of characters into larger meaningful units (words or phrases) and may thus facilitate reading. Conversely, the lightly spaced condition may still present a disruption to the normal reading of unspaced Chinese sentences due to the unusual presentation of the text. If this is the case, we might observe a slow-down (even if a mild one) in the lightly spaced condition as compared to the unspaced one.

Another feature of our study is that we consider all words in all sentences, rather than specifically selected lexical fragments of sentences. This enabled us to link comparisons of sentence reading times across experimental conditions to comparisons of word reading times across the same conditions. As demonstrated below, such links allow for a greater precision in achieving our goal of identifying sources of similarities and differences between different types of unspaced and spaced Chinese texts.

## Method

### Participants

Eighty-two undergraduate students (mean age: 19.7) from McMaster University participated in the study. Forty-one participants took part in Experiment 1 (mean age: 19.5), and the remainder participated in Experiment 2 (mean age: 19.9). They were all native speakers of Chinese with Mandarin (72), Cantonese (9) and Wu (1) being their home dialects. Although there are differences in accent, lexis and minor differences in grammar between dialects of People's Republic of China, thanks to the use of a logographic script and a unified writing system, written Chinese is said to transcend dialectal differences (Li, 2006). Moreover, all of our participants reported they were fluent speakers and readers of Mandarin. The mean time spent in Canada was 4.4 years, with a range of 0.5 to 16 years. All subjects had normal or corrected to normal vision. All participants received a course credit or a monetary compensation of 20 CAD for their participation.

### Apparatus

Participants' eye-movements were monitored using the SR Research Eye Link 1,000 system (Kanata, Ontario, Canada) at a sampling rate of 1,000 Hz. The participant's head was stabilized with a chin and forehead rest. Eye movements were recorded from the right eye only. The stimuli were presented using Experiment Builder software on a white background in NSimSun fixed-size font on the monitor with a 1,024 × 768-pixel resolution. The distance between the monitor and participant's head was 60 cm, and characters were the size of 28 × 28 pixels and the size of a space (in spaced conditions) between words was equal to one-character size. 1° of visual angle included about 1.5 characters.

### Materials and Design

#### Stimuli

We used all 100 sentences from Liu et al. (2013) and 120 sentences from Wang et al. (2015) where every transition between Chinese characters is associated with the percentage of raters who placed a word boundary in that position (see Supplementary Stimuli). Liu et al. (2013) collected their segmentation judgements from 142 undergraduate and graduate students in Beijing, whereas Wang et al. (2015) used a crowdsourcing method on the Crowd Flower platform from more than 120 raters who were all native speakers of Chinese. We operationalized this percentage as a word's segmentation probability. What probability threshold to choose for the insertion of spaces is a design decision that can influence the reading strategy in both the spaced and unspaced conditions, as well as the role of spacing and that of segmentation probabilities. No single choice of a probability threshold is optimal. For instance, limiting insertion of spaces to only high-probability transitions would reflect a very small fraction of segmentation preferences among readers whose natural consensus on word segmentation is around 64% (Liu et al., 2013). Moreover, that would only cover a small subset of cases in which readers have to make a segmentation choice. On the other hand, allowing spaces at most transitions, including ones that are viewed as valid word segmentation cues by only a small fraction of readers (i.e., low-probability transitions) will offer a greater sample of segmentation choices, but will make reading of spaced texts less naturalistic. A full investigation of the interplay between segmentation probability and spacing requires a series of studies in which the probability threshold for space insertion is systematically manipulated along a range. In this study, we implemented two extremes of segmentation probability as realized in our two conditions: a heavily spaced condition, where we inserted a space between characters if the segmentation probability of that transition was 0.05 or higher (i.e., if 5% or more of raters put a word boundary at that transition in the rating task); and a lightly spaced condition, where a space was inserted between characters if the segmentation probability was 0.90 or higher (i.e., if 90% or more raters identified it as a word boundary).

Effectively, in the heavily spaced condition spaces were only missing in the between-character transitions that were not considered a suitable word boundary by virtually any rater. We opted for a low-probability threshold for space insertion to make sure that our spaced condition is a true counterpart to the unspaced condition, where readers' decisions on how to segment characters into words are made based on both the low-and high-probability character strings. Also, as our literature survey above demonstrates, the case of spacing in high-probability character transitions is better studied, while the full inter-word spacing option in written Chinese is only used in a handful of studies (e.g., Inhoff et al., 1997). Even with our lax inclusion criteria of 5% in the heavily spaced condition, the median segmentation probability of spaced transitions in this condition was 93% (**Table 2**). Thus, most of the target transitions in the heavily spaced condition were supported by the consensus and the number of spaces was no more than a half of the number of characters in every sentence (see example in Table 1). The number of inserted spaces was obviously smaller in the lightly spaced condition, which had a 90% threshold of the raters' consensus as a spacing threshold, see Table 1. We reasoned that if spacing is beneficial for reading of Chinese, such a condition will create the best environment for the benefit to materialize.

**Table 1:** Example sentence with two spacing conditions and segmentation probabilities between words.

**Condition**	**Sentence**
Normal unspaced	中国拥有巨大的市场, 在游戏产业中无疑应当成为主导力量。
Heavily spaced	中国拥有巨大的市场, 在 游戏 产业 中 无疑 应当 成为 主导 力量。
Lightly spaced	中国拥有巨大的市场, 在游戏产业中 无疑 应当 成为 主导力量。
Segmentation probabilities between words	中国1.0拥有1.0巨大0.31的1.0市场,在0.88游戏0.62产业0.60中0.95无疑0.95应当0.90成为1.0主导0.52力量。
Translation	China has a huge market, and it should undoubtedly become a dominant force in gaming industry.

In the unspaced condition, sentences were presented in their conventional form, without spaces. A spaced counterpart (lightly and heavily spaced) was created for every original unspaced sentence. Examples of stimuli can be found in Table 1 below. Experiment 1 presented one group of readers with the unspaced and heavily spaced sentences, while Experiment 2 presented another group of readers with the (same) unspaced and lightly spaced sentences. In each experiment, two counterbalanced lists presented a mixture of unspaced and (heavily in Experiment 1 or lightly in Experiment 2) spaced sentences, such that every participant was presented with one of the lists and saw each sentence in only one format. Each list contained 110 spaced and 110 unspaced sentences. Sentences appeared on a single line, with a minimum of 19 characters and a maximum of 42 characters.

#### Procedure

Upon arrival, participants signed a consent form and were instructed to read sentences silently for comprehension. Yes/no comprehension questions appeared after roughly 30% of sentences. Participants were asked to answer “yes” by pressing “1” on the keyboard in front of them, and “no” by pressing the “0” button. After setting up the eye-tracker, a 9-point calibration was conducted. We required calibration accuracy to be below 0.5° of the visual angle to proceed with testing. If the validation procedure was not successful, the participant was removed from the study. Then, participants read six practice trials prior to presentation of the critical stimuli. Each trial started with a drift correction procedure, which was initiated with a dot placed at the location of the first character of a sentence. After finishing reading each sentence, participants were asked to fixate on a gray box in the lower right corner of the screen. Once the box was fixated for 200 ms, the screen was changed to display the next sentence. After the reading task, all subjects completed the LEAP-Q (Language Experience and Proficiency) questionnaire for every language they were fluent in (Marian et al., 2007). Since we tested readers of Chinese outside of China, this information was important to assess their proficiency in reading Mandarin, as well as their degree of exposure to other languages. The whole experiment lasted about 60 min. Both experiments had identical procedures.

### Variables

We considered effects of spacing and control covariates at the level of word and sentence. The unit of analysis at the word level was the interest area contained between two spaces in the spaced condition of each Experiment. We contrasted these interest areas with respective fragments of written sentences in the unspaced conditions of Experiments 1 and 2. In the heavily spaced Experiment 1 those interest areas were obviously shorter than in the lightly spaced Experiment 2. For simplicity, we label these interest areas “words” in all conditions. The word level used the following dependent variables: first fixation duration, gaze duration (summed duration of all fixations made on a word in the first pass, prior to a saccade to another word), and total fixation time (summed duration of all fixations on a word). First fixation duration and gaze duration are early measures of lexical access, while total fixation time is considered a cumulative measures of word processing. Joint consideration of these measures can point to the time-course of the spacing effect on word reading.

The sentence level analysis has sentence as a unit and recruited the following dependent variables: sentence reading times (i.e., the total time spent reading a sentence) and comprehension rate (rate of correct responses to comprehension questions). Sentence reading time taps into the amount of cognitive effort that subjects experience when reading spaced or unspaced sentences, while comprehension rate taps into the effect of spacing on comprehension. We also considered total saccade duration (summed duration of all saccades in the sentence) and total number of saccades per sentence, see rationale and analysis below.

#### Independent Variables

The critical variable was the experimental condition of spacing with three levels: unspaced (identical in Experiments 1 and 2), heavily spaced (Experiment 1) or lightly spaced (Experiment 2). For the word level analysis, word length in characters, word position in a sentence and position of a sentence in the experiment were included as controls. Because all our texts are identical–with a sole exception of spacing–we do not consider the many lexical predictors known to affect eye-movements, e.g., word frequency, predictability, and spatial density: these predictors are kept constant across conditions.

For the sentence level analysis, sentence length in characters (including spaces) was taken into account as a control. We also considered the position of a sentence in the experiment as a potential predictor of reading times at the sentence level. If found, such effects may indicate habituation to the unusual presentation in the spaced condition and perhaps development of a strategy toward using spaces as segmentation cues. With regard to individual differences, we considered years of education, reading comprehension in Mandarin and years spent in Canada to predict sentence reading times. We further tested interactions of these participant variables with spacing.

### Statistical Considerations

Durational dependent variables (measured in ms) showed skewed distributions and were log-transformed, as indicated by the Box-Cox test, in order to obtain a more symmetrical distribution and conform with the requirements of regression modeling. This is in line with recommendations from Baayen and Milin (2010). The comprehension rate scale was a distribution of zero and one, where zero stands for an incorrect answer and one stands for a correct response. Logistic regression was fitted to explore the effect of spacing on comprehension rate.

We used library lme4 version 1.1-19 (Bates et al., 2018) in the statistical software platform R 3.4.3 (R Core Team, 2017) to fit linear mixed-effects models to calculate the effect of multiple predictors on each dependent variable mentioned above. The model utilized sentences and subjects as random intercepts, which allowed us to examine systematic effects considering the variability across participants and testing items. We further modeled by-participant contrasts of spacing condition as random slopes. Since this step led to consistent failure-to-converge errors in regression models, we removed this random effect (Barr et al., 2013). The fixed effects in our models are described in the Independent Variables section above.

Our further model selection process involved fitting fully defined models (with independent variables as described above) and then back-fitting the model to retain significant fixed effects and obtain a final, best-fit model. Specifically, we used the likelihood ratio method for model comparison to identify whether removal of a predictor has led to a significant decrease in the model performance. Predictors that did not lead to such a decrease were removed with the exception of the critical predictor of experimental condition. At each step, no more than one predictor was removed, and the model was refitted; the process was iterated until removal of any predictor in the model (except for that indicating experimental condition) led to a significant loss in the model performance. Justification of this practice is outlined in Baayen et al. (2008). In consideration of space, we do not publish all regression models involved in the back-fitting process: these models are available upon request from the authors.

When fitting each model, in order to eliminate the influence of outliers, we also removed residuals that exceed 2.5 standard deviations, see Baayen and Milin (2010). The models in which critical predictors and interactions reached statistical significance are reported in the Supplementary Tables.

We chose to confirm our critical conclusions by estimating the amount of support for the null or alternative hypothesis by calculating the Bayes Factor. The Bayes Factor quantifies the ratio between the likelihood of the data under the alternative hypothesis and the likelihood of the data under the null. To estimate the Bayes Factor, we followed the procedure outlined in Masson (2011): we extracted the Bayesian Information Criterion (BIC) value for the target model and compared it to the BIC value of a model without predictors of interest (the “null model”). The Bayes Factor can be approximated as the natural exponent raised to the power of half the difference between the BICs of two models (see Masson, 2011). Following Jeffreys (1961), a Bayes Factor (BF) value below 1/3 indicates moderate support for a null hypothesis (and above 3 for the alternative) and those below 1/10 indicate a strong support for the null hypothesis (and above 10 for the alternative). We have also estimated the size of all critical effects by means of Cohen's *d* for a comparison of the two groups formed by experimental conditions.

## Results

In total, seven participants were excluded from Experiment 1. Data from two participants were discarded due to poor calibration (2,042 observations, 1.4%). Another three participants were excluded from the analysis due to excessive skipping rates (8,416 data points, 5.8%), one participant was excluded due to zero answers recorded when answering comprehension check questions and one more was removed due to an at-chance comprehension rate (7,588 observations, 5.2%). Similarly, analysis of Experiment 2 excluded two participants as none of their answers to comprehension questions were recorded and one more participant was removed due to an at-chance comprehension rate (6,396 observations, 7.3%), Thus, this left us with a pool of 72 participants for two experiments.

For word level analysis, after removal of a total of ten participants and sentence-initial and-final interest areas, we had a pool of 121,494 interest areas (83.03%) for Experiment 1 and 65,661 (75.5%) interest areas for Experiment 2. We further trimmed the bottom and top 1% of fixations from the distribution of total fixation time [Experiment 1: 1,211 observations (0.8%); Experiment 2: 1,948 observations (2.2%)]. At this point, this trimming resulted in 120,283 data points for Experiment 1 and 63,713 for Experiment 2. This full dataset was used to calculate the skipping rate, which was around 52% for Experiment 1 and 25% for Experiment 2. According to Chen et al. (2003) the probability of skipping tends to be much higher in Chinese readers than in English readers (42 vs. 20%). After removing skipped words, we had a total of 61,779 observations for both conditions (heavily spaced and unspaced) in Experiment 1 and a total of 46,656 data points for lightly spaced and unspaced conditions in Experiment 2 for word-level fixation time analysis. For sentence level analysis, after removing ten participants, we had a pool of 7,330 sentences for Experiment 1 and 8,322 sentences for Experiment 2. We further trimmed the bottom and top 2% of the sentence reading time distribution (629 trials, 4%). In total, Experiment 1 had 7,036 sentences and Experiment 2 had 7,987 sentences that entered sentence level analysis.

Below we begin with reporting the main effect of spacing on eye-movements at the word and then sentence level for each of the two experiments separately. Table 2 reports descriptive statistics for independent and dependent variables (see the Variables section).

**Table 2:** Descriptive statistics of independent and dependent variables across three conditions.

**Variable**	**Exp**	**Condition**	**Range**	**Mean**	**Median**	**SD**	**Range of log values**
N of trials			1:220				
Segmentation probabilities			0.06:1	0.75	0.93	0.30	
N of space-separated items in a sentence	1	HS	11:31	11.18	11	6.70	
	2	LS	5:22	7.24	7	4.50	
N of characters in a sentence			19:42	31.35	32	4.90	
Sentence reading time, ms	1	HS	1,030:8,554	3,158	2,795	1,473.90	6.94:9.05
		US	1,035:8,547	3,160	2,819	1,494.42	6.94:9:05
	2	LS	1,214:9,109	3,442	3,091	1,556.27	7.10:9.12
		US	1,216:9,110	3,511	3,178	1,589.36	7.10:9.12
First fixation duration, ms	1	HS	50:947	221.37	202	85.60	3.91:6.85
		US	50:976	231.93	213	91.51	3.91:6.88
	2	LS	51:995	246.76	223	105.46	3.93:6.90
		US	51:984	246.50	224	104.00	3.93:6.89
Gaze duration, ms	1	HS	50:976	236.40	208	106.61	3.91:6.88
		US	50:980	246.46	218	113.88	3.91:6.89
	2	LS	51:1,746	312.13	253	195.63	3.93:7.47
		US	51:1,794	312.24	254	195.47	3.93:7.49
Total fixation time, ms	1	HS	50:980	294.27	240	164.64	3.91:6.89
		US	30:980	310.67	254	173.86	3.91:6.89
	2	LS	51:1,793	438.23	344	303.64	3.93:7.49
		US	51:1,794	431.74	336	299.03	3.93:7.49
Total saccade duration, ms	1	HS	106:4,803	787.67	681	452.52	4.67:8.48
		US	106:4,580	730.00	625	432.41	4.66:8.43
	2	LS	68:6,482	743.59	562	652.28	4.22:8.78
		US	52:6,147	749.55	579	629.84	3.95:8.72
Total saccade number	1	HS	7:45	18.33	17	7.71	1.95:3.81
		US	7:45	17.30	16	7.33	1.95:3.81
	2	LS	7:60	17.74	16	8.72	1.95:4.09
		US	7:60	18.11	16	8.60	1.95:4.09
Skipping rate	1	HS	0:1	0.47	0	0.50	
		US	0:1	0.51	1	0.50	
	2	LS	0:1	0.26	0	0.44	
		US	0:1	0.23	0	0.42	
Years of education			10:23	14.02	13	2.16	
Reading comprehension score			4:10	8.67	9	1.43	
Years in Canada			0.5:16	4.42	3	3.81	
Mean accuracy for comprehension questions	1	HS	0:1	0.88			
		US	0:1	0.89			
	2	LS	0:1	0.89			
		US	0:1	0.89			

*HS, Heavily spaced; LS, Lightly spaced; US, Unspaced*.

The mean comprehension rate of two experiments was 87.5%, which indicates that participants generally had a good comprehension of experimental sentences. No difference in accuracy was observed between the heavily spaced and unspaced conditions and lightly spaced and unspaced conditions, (all *p*s > 0.626). Also, there was no statistically significant difference in comprehension scores between spaced and unspaced conditions in both experiments when participants' individual measures (e.g., years of education) were added as co-variates, all *p*s > 0.11 (models not shown, available upon request).

### Experiment 1: Heavily Spaced vs. Unspaced Conditions

#### Word-Level Analysis

We explored the effect of spacing on a word by fitting separate regression models to first fixation duration, gaze duration and total reading time on a word with spacing, ordinal trial number, number of characters in a word, and word position in a sentence as predictors. All measures of the word-level analysis showed a significant effect of spacing condition, where words surrounded by spaces were read faster compared to non-spaced counterparts (first fixation duration, β = −0.058, SE = 0.003, *p* < 0.001, gaze duration, β = −0.066, SE = 0.004, *p* < 0.001, total reading time, β = −0.097, SE = 0.005, *p* < 0.001). Thus, word level analysis showed a beneficial effect of heavy spacing. Detailed results of all three regression models can be found in Supplementary Tables 1A,B–3A,B.

#### Sentence-Level Analysis

We fitted a linear mixed-effects model to log-transformed sentence reading time as a dependent variable and spacing condition as a critical predictor. Sentence length (in characters) and an ordinal trial number served as controls. We observed a significant positive main effect of sentence length on sentence reading times, β = 0.084, SE = 0.011, *p* < 0.001: unsurprisingly, it took more time to read longer sentences. Total reading times for sentences appeared to be numerically almost identical across experimental conditions, 3,158 vs. 3,159 ms, (*d* = 0.001). This effect was not significant when controlling for other predictors, β = 0.007, SE = 0.008, *p* = 0.388. The Bayes Factor analysis indicated extremely strong evidence in favor of the null effect of spacing, BF < 0.001. Detailed results of both regression models can be found in Supplementary Tables 4A,B, 5A,B.

**Figure 1:**
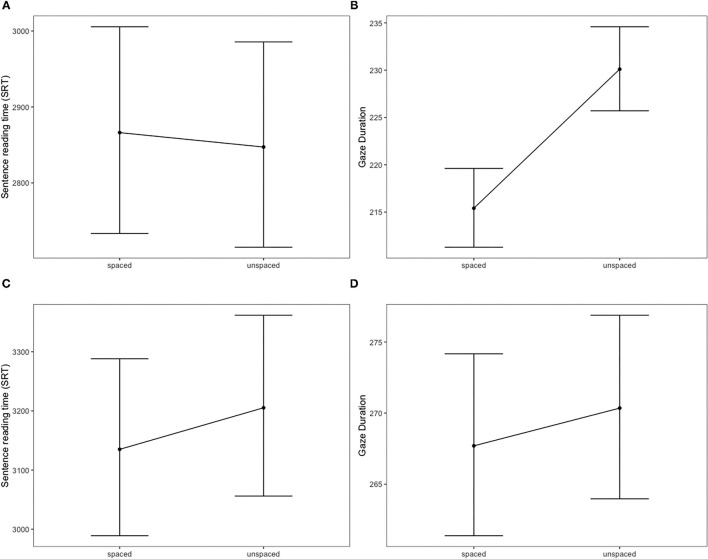
Top left **(A)** Partial effects of spacing (heavily spaced vs. unspaced) on sentence reading times, Experiment 1. Top right **(B)** Partial effects of spacing (heavily spaced vs. unspaced) on gaze duration, Experiment 1. Bottom left **(C)** Partial effects of spacing (lightly spaced vs. unspaced) on sentence reading times, Experiment 2. Bottom right **(D)** Partial effects of spacing (lightly spaced vs. unspaced) on gaze duration, Experiment 2. Error bars stand for 95% confidence intervals.

The results of the sentence analysis are consistent with previous studies, which mainly showed that reading times are statistically identical for spaced and unspaced sentences (e.g., Bai et al., 2008). Yet they may appear unexpected given that heavy spacing granted readers a small but significant advantage in speed at the word-level in Experiment 1 (see Figure 1 below). This word-level advantage was apparently canceled out by other factors when accumulated over a sentence.

We examined potential sources of this discrepancy. First, participants skipped more words in the unspaced condition than in the spaced one (51 and 47%, a small but a highly reliable 4% difference, χ^2^ = 234.12, df = 1, *p*-value < 0.001). Thus, even though each individual word was processed faster, more words contributed to reading times of the spaced sentences. A more drastic discrepancy, which may explain the null effect of spacing at the sentence level, was found in the measure of total saccade duration, or the sum of all saccade durations in the sentence. We found that total saccade duration in spaced sentences was longer than in unspaced sentences by an average of 58 ms (788 vs. 730 ms). This difference was confirmed as reliable in the mixed-effects regression model fitted to total saccade duration per sentence with sentence length and ordinal trial number in the experiment as a predictor (β = 0.077, SE = 0.017, *p* < 0.001). Spacing did not interact with sentence length (see Supplementary Tables 6A,B).

Saccade durations are rarely considered in studies of word reading. This is because the influence of inter-word saccades on word reading times is negligible as compared to fixation durations, and durations of intra-word saccades do not contribute to word reading times at all. However, in an experiment with sentences that have a median of 19 words, the number of saccades is considerable and saccade durations add up to a substantial proportion of sentence reading time (788 ms out of 3,158 ms or 25% in the spaced condition, and 730 ms out of 3,159 ms or 23% in the unspaced one). The accumulated total saccade duration is a factor that, along with other factors, appears to override the word-level advantage of spacing and lead to statistically identical reading times for spaced vs. unspaced sentences.

We further investigated whether the spacing-driven difference in total saccade durations is due to an inflation in the duration of individual saccades in the spaced condition or an increase in the average number of saccades (or fixations) in this condition, or both. The former option may arise because spaces introduce an extra character at every potential word transition, which also adds a disruption to the benefit of the parafoveal preview. Thus, spaced sentences might elicit intra-word saccades that need to be longer in amplitude and in duration. The latter option might stem from a smaller number of skips (and hence a larger number of fixated words and of inter-word saccades) in the spaced condition. The follow-up analyses revealed that average saccade duration was nearly identical in the two conditions (42.08 vs. 42.16 ms). Notwithstanding, the spaced condition came with a significantly higher total number of saccades per sentence than the unspaced condition (18.33 vs. 17.30). This contrast was confirmed as statistically reliable (β = 1.016, SE = 0.280, *p* < 0.001) in the regression model with sentence length as a control: spacing and sentence length did not interact (see models in Supplementary Tables 7A,B). In sum, the processing advantage seen in the spaced condition at the word-level is canceled at the sentence level, because spaced sentences elicit a larger number of saccades and fixations and—once the durations of those saccades are accounted for—come with the same total processing effort compared to their unspaced counterparts.

### Experiment 2: Lightly Spaced vs Unspaced Conditions

#### Word-Level Analysis

This analysis used the same dependent and independent variables as in Experiment 1. First fixation duration and total reading time analysis did not show any significant effect of light spacing, β = −0.004, SE = 0.004, *p* = 0.305, β = 0.001, SE = 0.008, *p* = 0.951, respectively. Interestingly, another eye-tracking measure, gaze duration, which captures time spent on a word during first pass reading, showed a beneficial effect of space at the significance threshold, β = −0.010, SE = 0.005, *p* = 0.041. Detailed results of the regression model can be found in Supplementary Tables 8A,B.

#### Sentence-Level Analysis

As in Experiment 1, mixed-effects regression model was fitted to log transformed sentence reading times including spacing condition, sentence length, and trial number as predictors. As expected, we observed a significant main effect of sentence length, β = 0.102, SE = 0.011, *p* < 0.001, meaning that longer sentences took longer to read on average. Contrary to the results of the first experiment, the difference in sentence reading times was significant and indicated a speed advantage for lightly spaced sentences, β = −0.022, SE = 0.007, *p* < 0.001. Sentences were read faster if spaces were inserted in highly probable word transitions (respective means 3,442 vs. 3,511 ms, with a relative difference of 2%; *d* = 0.05). The advantage of light spacing was then confirmed at both the word-level (gaze duration) and sentence-level: in both cases the effects were significant but small in size (see Figure 1). Detailed results of the regression model can be found in Supplementary Tables 8A,B, 9A,B.

By comparing the results from Experiments 1 and 2, we observe that the amount of spacing presented to our readers modulated their reading times. Heavily spaced sentences were read in the same amount of time as unspaced ones (Experiment 1), whereas Experiment 2 showed that spaces can bring advantage in reading speed when they are placed only at highly probable word transitions. In other words, spaces are only advantageous when introduced in positions where the majority of readers agree to place a word boundary.

Similar to Experiment 1, we further explored how the word-level findings link to sentence-levels ones, looking at skipping rates, and total saccade number and their duration in Experiment 2.

Skipping rate for the unspaced condition was 23.3%, while for the lightly spaced condition it was 26.0%. A chi-square test showed that this difference is statistically significant (χ^2^ = 60.32, df = 1, *p*-value < 0.001): there were more words skipped in the lightly spaced condition. Thus, more words contributed to the reading times of the unspaced sentences, which partially explains the inflated sentence reading times in that condition. Additionally, we explored if the number of saccades and their duration contributed to shortened sentence reading times in the lightly spaced sentences. Total saccade duration per sentence did not show any significant difference between lightly spaced and unspaced sentences (β = −0.023, SE = 0.015, *p* = 0.130). Although the number of saccades was numerically smaller for spaced sentences (mean: 17.74 saccades for spaced, and 18.11 for unspaced), regression analysis revealed only a marginally significant difference (β = −0.447, SE = 0.247, *p* = 0.071). To conclude, it was mainly the shortened fixation durations on words and a higher skipping rate in the lightly spaced condition that brought the advantage in sentence reading times to this condition over the unspaced counterpart.

We further examined the potential role of individual differences in the participants' education level, subjective assessment of Mandarin reading comprehension or duration of stay outside of China. None of these measures turned out to be predictive. Years spent in Canada or years of education did not show any effect on word or sentence reading times in Experiment 1 or 2. Higher subjective evaluation of reading comprehension was associated with shorter sentence times (*p* = 0.041). Critically, none of the measures modulated the effect of spacing on either word or sentence reading times.

## Discussion

Chinese does not have overt visual markers to separate words in a sentence, and the very notion of a word in this language is debated. There is no definitive consensus between Chinese readers on word boundaries, and their decisions on how to segment words in a sentence are contingent on a number of syntactic and semantic factors (Liu et al., 2013). This has led researchers to the question of how readers of Chinese segment a continuous sequence of characters into processing units and whether word units have a psychological reality in Chinese. A common approach to this question, which we also followed, is to artificially introduce spaces into naturally unspaced sentences. Previous research on the effects of spacing in Chinese sentences gave rise to mixed conclusions. Reports vary in whether these effects are facilitatory or inhibitory at the word level, and whether they exist at the sentence level (see the Introduction). Furthermore, while statistical probabilities of transitions between characters have long been recognized as a factor influencing mental segmentation of Chinese sentences, these probabilities have only been manipulated in a handful of studies (Yen et al., 2012; Zang et al., 2016) and, to our knowledge, not in conjunction with spacing manipulations.

Our study offered an examination of the effects of spacing on reading Chinese sentences by comparing natural unspaced sentences with counterparts that were either lightly spaced (spaces only at high probability transitions) or heavily spaced (spaces at every probable transition). The main goal of our study was to add to the currently incongruous body of evidence about the role of visual cues to lexical segmentation in Chinese reading by investigating the role of segmentation probabilities in reading artificially spaced text. We pursued this question by recording eye-movements in a sentence reading study in Chinese where participants read either conventional unspaced sentences or their spaced counterparts for comprehension. We also aimed at pinning down the specific sources of similarities and differences between spaced and unspaced texts by linking word-reading and sentence-reading times.

### Effects of Spacing in the Heavily Spaced Condition

The central result of Experiment 1 was that heavily spaced sentences and sentences without spaces took identical time to read. This is surprising, since heavily spaced sentences were spatially longer than their unspaced counterparts and should take a longer time to read. Nevertheless, this result is consistent with previous studies, which mainly showed statistically identical reading times for spaced and unspaced sentences (e.g., Bai et al., 2008). The word-level analysis showed that eye fixation durations became shorter when words were demarcated with spaces. All measures of early and late processing (first fixation duration, gaze duration, total fixation time) showed a small, but significant facilitatory advantage of having spaces as visual cues. This pervasive effect conflicts with Bai et al.'s (2008) argument that the segmentation into words appears not to happen at early stages of processing. We believe that the small effects on early eye-movement measures emerged as reliable in our study due to the higher statistical power of our dataset (see Brysbaert and Stevens, 2018, for recommended sample sizes).

Although we observed *shorter* eye fixation durations on individual words in the spaced condition, sentence-level analysis showed that this advantage was completely over-ridden at the sentence level: sentences with spaces and without spaces took identical time to read. We argue that partial explanations for this reversal come from the processing costs of spacing that are not noticeable in individual words but accumulate and become noticeable in sentence reading times, and especially the inflated total duration of saccades in the sentence. Total saccade duration, defined as a summed duration of all saccades in the sentence, accounted for about 25% of total sentence reading time in both conditions and was 58 ms longer on average in the spaced rather than unspaced sentences. We highlight the utility of total saccade duration as a measure that is largely overlooked in the studies of sentence or passage reading.

To our knowledge, this is the first study that attempts to explain contradicting results in the previous literature, which show word-level advantage of spacing but fail to do so at the sentence level. To reiterate, we found that a larger number of saccades and other factors, including a reduced skipping rate, in spaced sentences appear to inflate sentence reading times to an extent that cancels out the slight word-level advantage. In sum, when overt visual cues for word segmentation are inserted at almost every transition where segmentation is possible (though not always very probable), spacing is not a cue that increases reading efficiency in Chinese. It also does not lead to improved reading comprehension.

### Effects of Spacing in Lightly Spaced Condition

Experiment 2 draws a different picture. In sentences where spaces were placed only at highly probable word transitions, a beneficial effect of spacing on sentence reading times was demonstrated. Additionally, word-level analysis showed a beneficial effect of spacing through shortened word reading times (gaze duration) and increased skipping rates in the spaced sentences. That is, both sentence-and word-level analyses showed that spaces inserted only where the majority of readers expect a word boundary is demonstrably advantageous for reading Chinese. The observed difference between heavily and lightly spaced conditions in our Experiments 1 and 2 may partly explain discrepant findings in the earlier literature. The magnitude and direction of the spacing effect is contingent on the prevalence of spacing and, even more so by the probability of the character transition interrupted by a space as a word boundary. Since these probabilities were not systematically controlled in most earlier studies using spacing, divergence in results across studies is expected. In sum, the prevalence of spacing and its allocation in a sentence does indeed modulate sentence reading times: spaces at highly probable word boundaries lead to a small (around 2% of relative difference) but reliable advantage.

The present study contributes to the existing body of knowledge on effects of spacing in the following ways. First, results from both experiments indicate that the effects of spacing are selective and contingent on the prevalence and exact positioning of spacing in the Chinese text. Contrary to some previous research, this study shows spacing to be a cue beneficial to both the Chinese word segmentation process and, in one of conditions, for sentence level processing. However, spacing only becomes beneficial when readers find spaces at suitable word boundaries, and even then, the processing advantage is minute. These findings demonstrate that segmentation probabilities are an important yet relatively under-studied factor to consider in research of Chinese reading.

Second, our joint analyses of reading times at the word and sentence level enabled us to uncover reasons for similar or discrepant processing times across experimental conditions that much earlier research left unexplained. For instance, it highlighted the role of saccades, which increase in number and duration with the prevalence of spaces and can cancel advantages conferred by spacing as a segmentation cue at the word level. We advocate the use of a largely neglected saccade analysis in eye-tracking reading studies as a useful tool for studying reading behavior.

Finally, our investigation of two extremes of segmentation probability as criteria for the placement of spaces across all sentences suggests that spacing is not an effective segmentation cue in Chinese reading. In either the heavily or lightly spaced conditions, the advantages that spacing confers at the word level, if any, are small in size and either completely canceled out at the sentence level or diminished to the effect size of no practical importance. Most likely, further investigation of spacing at less extreme points of the probability scale will lead to a similar result. It is plausible that other methods of guiding attention through Chinese sentences (e.g., coloring or highlighting segmentation boundaries) will not lead to the presently observed increased difficulty of saccadic planning and enable the word-level advantage in processing effort to propagate to the sentence-level. An investigation that combines the use of less invasive segmentation cues with probabilistic characteristics of character transitions is a promising avenue for future research.

## Data Availability Statement

The raw data supporting the conclusions of this article will be made available by the authors, without undue reservation, upon request to the corresponding author, Gaisha Oralova, oralovag@mcmaster.ca.

## Ethics Statement

The studies involving human participants were reviewed and approved by McMaster Research Ethics Board. The patients/participants provided their written informed consent to participate in this study.

## Author Contributions

GO and VK contributed equally to the data analysis and manuscript write-up. GO was responsible for the experimental design and programming, collection and cleaning of the data. Both authors contributed to the article and approved the submitted version.

## Funding

GO's contribution was supported by Cognitive Science of Language Graduate Scholarship, McMaster University, The Elder Family Graduate Award (2017–2018) and by the Social Sciences and Humanities Research Council of Canada Doctoral Fellowship 752-2019-1264 (2019–2021). VK's contribution was partially supported by the Ontario Early Researcher Award (Kuperman, PI), the Canada Research Chair (Tier 2; Kuperman, PI), the Social Sciences and Humanities Research Council of Canada Partnership Training Grant 895-2016-1008 (Libben, PI), and the Canada Foundation for Innovation Leaders Opportunity Fund (Kuperman, PI).

## Conflict of Interest

The authors declare that the research was conducted in the absence of any commercial or financial relationships that could be construed as a potential conflict of interest.

## Publisher's Note

All claims expressed in this article are solely those of the authors and do not necessarily represent those of their affiliated organizations, or those of the publisher, the editors and the reviewers. Any product that may be evaluated in this article, or claim that may be made by its manufacturer, is not guaranteed or endorsed by the publisher.

## Footnotes

^1^We thank the anonymous reviewer for raising this point.
