# Supplementary material for: Effects of Spacing on Sentence Reading in Chinese
Source: Front Psychol. 2021 Nov 10;12:765335. doi: 10.3389/fpsyg.2021.765335 (PMC8631542; doi:10.3389/fpsyg.2021.765335)
Supplement: Supplementary file 1 [file Data_Sheet_1.docx]

| **Supplementary materials: Stimuli**  Please note, all stimuli sentences were selected from two sources listed below:  Wang, S., Huang, C. R., Yao, Y., & Chan, A. (2015). Create a Manual Chinese Word Segmentation  Dataset Using Crowdsourcing Method. In Proceedings of ACL-IJCNLP 2015 (pp. 7–14). Retrieved from <https://aclweb.org/anthology/W/W15/W15-3102.pdf>  Liu, P. P., Li, W. J., Lin, N., & Li, X. S. (2013). Do Chinese readers follow the national standard rules for  word segmentation during reading? PloS one, 8(2). |
| --- |
| 1. 尽可能弄清楚这个问题的原因和它同其他问题错综复杂的关系。 |
| 1. 在她们的身旁就是宽阔的交通要道，各种车辆在浓浓的黑烟中飞驰。 |
| 1. 面容慈祥的迪斯尼先生正拉着可爱的米老鼠的小手向朋友们挥手致意。 |
| 1. 有一个破坏力与落在日本广岛的原子弹相当的大陨星石将袭击美国西部。 |
| 1. 所以中国才从未出现过可以和吉川幸次郎媲美的日本文学研究家。 |
| 1. 对一些冤假错案要果断地依据法律来处理。 |
| 1. 五角大楼的新保守主义者想让民主像野草一样遍地生长。 |
| 1. 原则清晰地印在他们的大脑里并使他们能巧妙地与对方有计划的周旋。 |
| 1. 一个人只有当他专注于某件事时，他才会在这方面取得成绩。 |
| 1. 他不知道自己为什么也积极地加入这支追逐的队伍。 |
| 1. 要知道，只有阶级敌人才会这样无情地污蔑攻击我们的干部队伍。 |
| 1. 近两年几乎在舞台上消失的实力派歌手张咪突然出现在首都新闻界。 |
| 1. 实际上，石油也是由死亡的小生物转化来的。 |
| 1. 道德的标准很不一样，比如有人说买卖的婚姻是不道德的。 |
| 1. 中国拥有巨大的市场，在游戏产业中无疑应当成为主导力量。 |
| 1. 经过研究，他发现造成这种破坏的元凶是水中产生的一种气泡。 |
| 1. 天气图上密密麻麻地填满了不同的天气符号，这些符号都是有根据的。 |
| 1. 即使是这样，我们的残疾人运动员还是努力地投入了自己的青春生命。 |
| 1. 新兴的工业城市一开始就具有与西方不同的现代气质。 |
| 1. 朋友们都纳闷我是不是天生就长了一个中国情结。 |
| 1. 爱国将领与士兵勇敢地为了国家和人民而坚持抗日斗争。 |
| 1. 不同的政治派别似乎是同时地提出了大体相同的口号，这不是偶然的巧合。 |
| 1. 台湾的孩子们每到暑假往往被繁多的补习班所包围。 |
| 1. 冯敬义很希望这些讨厌的客人赶快离开他的房子。 |
| 1. 我曾为他们的判决竟同丘吉尔和杜鲁门的口吻相同而大为惊奇。 |
| 1. 因为他本来就很喜欢这种粗俗的诙谐，觉得它只不过是简单的玩笑罢了。 |
| 1. 这一古老的仪典已经失传很多年直到近些年又如雨后春笋般冒了出来。 |
| 1. 有许多种声音不断地叫着喊着，有许多只手慌乱无措的挡着拦着。 |
| 1. 甲状腺不能制造出足够的甲状腺素满足人们正常生活和生长发育的需要。 |
| 1. 他可以写一部小说，或在一部小说中简单地写一段关于天气寒冷的文字。 |
| 1. 她在我的生活中消失了这样久以后，现在重新站在了我的面前。 |
| 1. 一月八日，数千名中外游客兴奋地观赏着兵马俑。 |
| 1. 经过长时间的谈判，澳门总督最后终于无奈地接受了工人们的条件。 |
| 1. 一台台分体式空调器随着升降机和自动输送带正在不停地被装上汽车。 |
| 1. 继在半决赛中轻松击败古巴队后，意大利队在决赛中斗志昂扬高歌猛进。 |
| 1. 自从海底下那个难忘的片刻之后，他就不曾再抱过她。 |
| 1. 查扣八万美元大案是大连海关空港至今最大一起货币走私案。 |
| 1. 只有生他养他的那一方土地和土地上善良的那一群人，才能使他激动不已。 |
| 1. 年逾古稀但健康的他每日徜徉在由体育藏品所构成的温馨空间里。 |
| 1. 每个人都想成为王中王，但你不得不认真地一步一步来完成。 |
| 1. 佛塔、玉雕等文物精品再现了十八世纪中华文化的伟大和博大精深。 |
| 1. 全场希腊观众为东道主选手响亮的助威声终于变成热烈的掌声。 |
| 1. 在财会改革之后进行的是税制改革，紧锣密鼓声中新税制逐步替代了旧税制。 |
| 1. 刘维佳是去年一月举行的第五届全国城市运动会中优秀的年轻运动员。 |
| 1. 从国家需求考虑，上海硫酸厂接下了艰巨的生产任务。 |
| 1. 这山里娃子交了桃花运，竟有一位中尉女警官愿意和他结为伉俪。 |
| 1. 中国在短短十几年中取得了让全世界人震惊的巨大成就。 |
| 1. 出席这次会议的七国领导人在开幕式发言中都坚定地发出和平的呼唤。 |
| 1. 他要求侨联要努力保持长期以来形成的优良传统，积极探索联系侨界、服务侨界。 |
| 1. 虽然是第一次参加奥运会，这位20岁的姑娘已把目标放在夺金牌上。 |
| 1. 虽然新中国百废待兴，中国政府和人民仍无私地向越南人民伸出了援助之手。 |
| 1. 都灵奥组委还特意地在都灵市中心区建了两个展厅。 |
| 1. 他为观众们表演了精彩的少林童子功，一招一式有板有眼。 |
| 1. 透过那宁静悠徐的表面现象，你可以感到一种压抑的紧张气氛。 |
| 1. 中国的茶叶贸易正面临着历史上最大的严峻挑战和强大冲击。 |
| 1. 华东沿海农民朴实的精神风貌给来访者留下深深的印象。 |
| 1. 最新的研究结果表明经常玩积木可以更容易开启宝宝的智力。 |
| 1. 这次收购的是一家固定资产与自己相当的著名企业，因此格外引人注目。 |
| 1. 大量高标准的使用金箔，将是故宫再现辉煌的重要基础之一。 |
| 1. 但是她的力量和勇气却是长时间艰苦的磨炼的结果。 |
| 1. 报喜不报忧早已成了当今中国官场上平常的一件事情。 |
| 1. 临泉县公安机关竟然敢公然地编织谎言，逮捕前往上访的农民代表。 |
| 1. 大家都为阿力紧张，幸运的是最终阿力艰难地在浓雾中抵达东峰顶。 |
| 1. 此影片展示了日本平安时代中期宫廷贵族的激烈的权势之争。 |
| 1. 浙江经济的快速发展在于生活在这块土地上的平民百姓坚韧的创新精神。 |
| 1. 中国女乒的第三号选手牛剑锋幸运地获得了奥运会入场券。 |
| 1. 随着城市迅速地变大和变美，城里人的生活水平也在不断地发生着变化。 |
| 1. 经济全球化和世界科学技术快速的发展使知识产权保护面临新的挑战。 2. 在新的历史时期，政府希望中国红十字会继续传承红十字精神。 |
| 1. 掌握知识与发展智能是智育的两个关键的重要组成部分，不能有所偏废。 |
| 1. 中国经济的发展使九年代的外国商人看到了在大陆市场销售产品的前景。 |
| 1. 与其他机器人相比，这种机器人在行走环境复杂的室外更不容易跌倒。 |
| 1. 导演采用大量的镜头变换技法并将三部电影巧妙地衔接在一起。 |
| 1. 实践有助于我们了解这个理论的内涵和取得这个认识成果所付出的巨大代价。 |
| 1. 林肯登上权力的顶峰，在很大程度上得益于他那非凡的演说才能。 |
| 1. 这静谧的田家村庄遗憾的是缺少点鸡鸣犬吠的声音。 |
| 1. 某些部门却不能或者说不敢站出来正当地维护劳动者的权益。 |
| 1. 她有时候也生气地和他吵几句嘴，不过，在事后一想，越吵嘴便相隔越远。 |
| 1. 黑河作为中国开放的前沿必将全面地迎接激烈的挑战。 |
| 1. 正因多种经营迅猛发展才有了乡镇企业的进步和社会经济事业的全面发展。 |
| 1. 为能早日出成果，他们每天在实验室努力地工作十多个小时。 |
| 1. 她们骑自行车从二十公里外选购了漂亮的油漆并自己动手粉刷墙壁和书架。 |
| 1. 悲剧和喜剧交融在一起，赋予了作品丰富的艺术魅力。 |
| 1. 对经济增长和就业具有显著的影响的服务业占美国经济活动的80%以上。 |
| 1. 那个单位里有很多资格和地位跟他相似的老同志。 |
| 1. 这一刻的休息我觉得很舒服，遗憾的是我们不能彼此交谈。 |
| 1. 兄弟俩带着在战争炮火中侥幸保存下来的荷兰奶牛来到陕北的牧场。 |
| 1. 这些规定的目的就是让好的产品能够畅销并受到市场尊重。 |
| 1. 英国人卡文迪许是世界最伟大的实验科学家之一。 |
| 1. 也许在有人看来，这不过是个不值得考虑的次要问题，但事实是它至关重要。 |
| 1. 目前这些经纪人都来自华尔街上著名的顶级投资银行和金融机构。 |
| 1. 此次俄罗斯方面将派出十二匹良驹参赛，门票起价为1800卢布。 |
| 1. 他们拍摄的是诸如博物馆等普通的旅游景点，根本不是“敏感”目标。 |
| 1. 中国有令人自豪的历史文化，也有足以让当今世界震惊的经济发展速度。 |
| 1. 这篇小说抛弃了非常关键的要素只是一味地去追求情节的紧张惊险。 |
| 1. 虽然满天星掀起的风波还没有平息，但它给人们留下了许多有益的思考。 |
| 1. 有家私立诊所，一夜之间便迅速成了宠物医院，患者络绎不绝。 |
| 1. 联盟汇集了台湾著名的女性专才，成员以企业家为主。 |
| 1. 改革开放后不久，中国人民独特的勤劳和智慧很快使我国的经济腾飞。 |
| 1. 由于零售店面都不大，销售的商品却相当多，该怎么陈列，的确很费心思。 |
| 1. 前面按动摄影机快门的声音，使苦于高山反应的我们陡增劲头。 |
| 1. 后来，果然是件好事，因为走失的马带回来一群野马，老先生因而发了一笔横财。 |
| 1. 翌日早报的地方版，报导了镇上的色狼事件，一位小学女学童惨遭侵袭。 |
| 1. 我能强烈感到自己手心不断冒出冷汗，却只能竭尽力量牵动嘴角露出惨然苦笑。 |
| 1. 她把饼盒放进车里，站在车门边喘气，好像再也走不动了。 |
| 1. 想不到，山顶上非常平坦，碧绿的草地上开满了不知名的花，风景非常的幽美。 |
| 1. 第三世界国家将能逐渐提高生活水准，并至少能支付他们巨额外债的利息。 |
| 1. 天色完全黑了，商店的招牌也此起彼落的亮了起来，家家户户的灯火，发出温暖的光芒。 |
| 1. 任何天才，他一生作品中，真正有价值的只有四五件，其余只是他日常生活的填充物。 |
| 1. 当一位门外汉被雇用时，大家都倾向于等待救星出现。 |
| 1. 想起几个月以前，刚到这个新环境的时候，我还是一个带着美国口音的老外。 |
| 1. 知识和武器是这弱肉强食的世界里主持公道无可替代的两件法宝，你不能有片刻忘记。 |
| 1. 你可以和家人或朋友组队，一起在地上用粉笔临摹名画，或者只是在旁观赏。 |
| 1. 小马长大了，老马就请黑狗来教小马跑路，请山羊来教小马爬山，请黄牛来教小马拉车。 |
| 1. 当开始尝试写作论文，大家都知道，论文大纲是有典范可循的。 |
| 1. 最妙的是，不榨汁的时候，合上盖子，就可阻断果汁，并可防止切口氧化。 |
| 1. 宋代有一种以讲故事为职业的说书人，他们说书的时候所用的脚本就是话本。 |
| 1. 在家人、公司和张小燕的支持下，那段低潮终于烟消云散。 |
| 1. 在一定的技术下，每一劳动者有效使用的资本，有一最大的限量。 |
| 1. 这个奖项无疑是对他天王地位一次专业性的肯定和褒奖。 |
| 1. 现在我就快要成功了，绝不能在紧要关头自乱阵脚，一定要把这出戏演完，再撑个半小时。 |
| 1. 要是您买的东西有问题，也可以把发票跟东西一起拿来，我们就给您换。 |
| 1. 我这个老毛病总是改不了，只会在口头上卖弄一下，不采取行动，又能期望什么呢。 |
| 1. 风靡华人社会的歌星邓丽君在今年五月八日走完她短短的四十三年人生路。 |
| 1. 美元作为国际间主要关键通货，其币值却极不稳定。 |
| 1. 僧侣为人指点迷津，寺庙里，常有穿牛仔裤的年轻人在佛像前打坐沉思。 |
| 1. 我仰望着屋顶，透过那些裂缝和破瓦，云天居然历历在目。 |
| 1. 桥头边的榕树下，我看着这一摊因发电机的噗噗声而益发显得人气兴旺的小吃摊。 |
| 1. 为了消除种族间的差异，进入佛门的出家人，第一件事也是剃光头发。 |
| 1. 未能赶上当班车的旅客，不必站长签章，即可补空位上车或换乘其他车辆。 |
| 1. 不过，茶叶商都知道台湾红茶品质好，以品质考虑，我认为这个价格是合理的。 |
| 1. 在种种事件真相的背后，有一只庞大的资本义义幕后黑手，在从事着阴险狡诈的阴谋。 |
| 1. 她从小生长在富贵之家，受到父母的宠爱，所以脾气大，个性刚强，非常任性。 |
| 1. 我们先走进一个教剪纸的屋子，我和妈妈各剪了一个人像，带回家做纪念。 |
| 1. 与刚进大门时黑暗昏沉的环境相反，画室里的光线明亮充足，空间洁白宽敞。 |
| 1. 生意做垮后，他在人多的路口卖拖鞋，警察开的罚单多过整个下午卖的钱。 |
| 1. 我故作痛苦地把眉头一皱，作欲呕状，她吓得怔住了。 |
| 1. 每年夏天西瓜成熟的时候，远远看过去，一片碧绿，真是美丽。 |
| 1. 事实上，精品的音响着实迷人，其音色有如上好的酒，香水或珠玉。 |
| 1. 这时太阳更毒，一路上都是上坡，整个车队行进缓慢，大家都被烤得头晕脑胀。 |
| 1. 人类会不会似早期的恐龙一样，因为天灾而遭灭亡这个问题一直是好莱坞感兴趣的题材。 |
| 1. 某些蔬菜油等成分都可能刺激皮肤，引起粉刺；所以青春痘患者最好不要使用化妆品。 |
| 1. 他更语出惊人的表示，原本受害住户要出面检举，但被黑道威胁，车窗遭砸毁，所以不敢出面。 |
| 1. 途中经过一处市集，非常热闹，白菜又大又漂亮，这里的水质好从菜的发育良好就可看出来。 |
| 1. 以它的施工进度，大概最晚下学期，这个谜底就可以完全揭晓了。 |
| 1. 这是两种不同的农耕方式，但两种的谷物生产总量约略相同。 |
| 1. 三位金童神箭同步拉弓，把弓箭拉到最适合的张力，然后射向红通通的太阳。 |
| 1. 嘴角叼着烟，男孩把煮好了的咖啡倒在我手上的咖啡杯和那个有缺口的碗。 |
| 1. 世界贫穷地区的孩子，饥饿而死，而世界上尚有许多富人过着挥霍地球资源的生活。 |
| 1. 黑心皇后听了之后，大为震怒，便指派手下去迫害白雪公主。 |
| 1. 救援队员利用观察器就可以把探头探测到的地方看得清清楚楚。 |
| 1. 不断在前线杀人打仗，她早就想逃，但叛军看人看得很严，很难有机会。 |
| 1. 杨业作战勇猛无比，外号杨无敌，宋太宗很赏识他，命他担任北边守将。 |
| 1. 去年流行歌坛出现另一匹让人刮目相看的黑马动力火车，短短的几个月唱片大卖七十万张。 |
| 1. 宇宙的事物都有用处，即使小如一滴水，不仅可以止渴、洗澡、洒树浇花也很好呀。 |
| 1. 进了店里，我向老板要了两碗汤圆，就径自坐在座位上，若无其事地翻着桌上的报纸。 |
| 1. 读书完全是靠自己自动自发，有时间读书，叫做福气，多读书就是自强。 |
| 1. 水产玉与沙产玉则是由山上冲刷入河床的玉石，块头不大，质地精美。 |
| 1. 他躲在附近远房姑家的废弃鸡寮内，七天来只吃了十二颗龙眼，入夜才出来找水喝。 |
| 1. 酒精将成为明日能源的新星，而小小的酵母菌也将成为人类解决能源危机的希望所冀。 |
| 1. 一看到美丽、温柔的大海，我立刻飞也似的冲进它的怀抱，让清凉的浪花打在我身上。 |
| 1. 一到河边，我们都迫不及待的想下去玩，于是把拖鞋往河边一放，人就冲进河里去了。 |
| 1. 这也是今日塞纳河两边留有许多绿色大铁箱贩卖旧书的由来。 |
| 1. 他就捉了许多萤火虫，放进一个纱袋里，靠萤火虫发出来的亮光，他才可以读书。 |
| 1. 鞋子是足部保护的第一道防线，鞋子的发明设计在最原始的用意就是保护足部。 |
| 1. 老师也以为他天资比较差，脑筋迟钝，所以每天只教他半页书，总共不过几十个字。 |
| 1. 红色的金鱼游水很慢，游水的样子也不好看，可是头脑很好，一天能想很多事。 |
| 1. 农人有一双黝黑的手，这双又粗又黑的手，在田里卖力的工作，我们才有米饭和果菜吃。 |
| 1. 虽然泰国的变性人已经跨越了性别与角色的界线，但仍有许多的禁区尚待开启。 |
| 1. 我是清华园的留鸟，一年四季抬头望天空都有机会看到我们成群的在高空飞翔。 |
| 1. 他还不只是在早上洗澡，跟朋友碰面前，他都要洗得香喷喷的，再喷上香水才肯出门。 |
| 1. 一定是连日疲累，方才淋了雨，又抽大麻睡着了，因此受了风寒。 |
| 1. 由于火灾损失惨重，烧出了许多安检上的问题，也烧出了消防人员的风纪问题。 |
| 1. 他终于完成了这个梦，于是他带着人们，乘着小船划向了那座冰山，不再回来。 |
| 1. 甘肃省武威县自唐代起即为西北第一重镇，以大砖筑成的城墙十分壮观。 |
| 1. 东尼就把所有女孩子的约会都取消了，电视台的节目也都交给助手全权处理。 |
| 1. 我们就毫无节制的吃，直到涨饱了，竟看到侍者拿着一串串烤肉进来，才知这才是主菜。 |
| 1. 日常的衣食住行，没有一样不是别人花费了许多心血，才使我们得到了方便。 |
| 1. 一代代读书人对书的企盼，对知识的渴求，就像这个楼阁，都有一段苍凉而神圣的心路旅程。 |
| 1. 停水、跳电是常有的事，经常洗澡洗到一半，不是没冷水就是没热水，使学生非常不方便。 |
| 1. 虽然我不懂得歌词，但其优美的旋律和动人的歌声也能深深的感动我。 |
| 1. 我现在正办自费留学，只等在黑市上换够了美元，我就与各位告辞了。 |
| 1. 没有任何媒体可以真正看穿她的思维，甚至包括同为女性的我们都有无数的问号和臆测。 |
| 1. 李文秀双手合着鸟儿，手掌中感觉到它柔软的身体，感觉到它迅速而微弱的心跳。 |
| 1. 这位司机有个小苦恼，客人遗忘在车上的钱包，他挣扎着到底要拿去招领还是自己扣下来。 |
| 1. 黑夜的丛林里，红色的跑车像深夜出没的猛兽，缓缓向猎物推进，最后停了下来。 |
| 1. 她只是怀念追求时期与蜜月当中的温馨与亲切，忘记了婚后现实的环境。 |
| 1. 在中美洲兴建一条沟通两大洋的运河，自十六世纪以来即是许多人的梦想。 |
| 1. 我在闷热的新加坡码头上了快艇，船舱内凉凉的空调催人欲眠，不小心打了个盹。 |
| 1. 你若做得好，别人做得坏就显出来了；你唱高调，别人就显得低调了。 |
| 1. 大都市人口集中，楼房耸立，空气及河水污染得浑浊不堪。 |
| 1. 这个小插曲大家知道就好，犯不着挂在心上，也不必随意张扬。 |
| 1. 直到有一天，她在天桥上看到一幅巨大的广告看板，画里人物竟是她梦境中的书生。 |
| 1. 我有一个对这个命题十分美妙的证明，但是因为这里空白太小了我无法写下。 |
| 1. 离开留园，天色渐晚，我央求友人一定要带我到寒山寺一趟，他不忍拒绝，于是我达成了心愿 |
| 1. 他暗暗好笑，这样的防范，最笨的杀手也能避过的。 |
| 1. 黛丝用她那不死不活的女声平和地道︰拉利先生，三分钟后将到达都灵市，请减慢车速。 |
| 1. 看着一群群洁白的海鸥在轮船上方振翅飞翔，姑娘慢慢地停止了哭泣。 |
| 1. 老师教大家先把火点燃，再把玉米放进瓶盖，用铁丝把瓶盖绑好。 |
| 1. 这座滨临波罗的海的大城，十八世纪初由彼得大帝以西方城市为蓝本一手打造。 |
| 1. 巴格达电台引述卫生部长沙兰的声明说，缺乏奶粉也造成儿童健康情况的恶化。 |
| 1. 沙滩上满溢人群，四处是欢乐的笑声，慵懒的神态写在每个人的脸上。 |
| 1. 近几年来民间兴起创办书院的风气，以对成人讲经为主，鼓励人重新亲近老祖宗的智慧。 |
| 1. 我妈有时看他来了会流露出厌烦的表情﹐好像是家里手头比较紧﹐可他来了总要给点 |
| 1. 它是作家从事创作的基础，也是读者了解作家的注脚和窗口。 |
| 1. 各灯区的花灯只有晚上才会发光，绚丽的灯光与天上的明月相互争辉。 |
| 1. 在上海念了五六年书，换了三个学校，一张文凭也没拿到，而老家的情形更令人灰心。 |
| 1. 小刚对她的苦心全不理解，不但不配合着往前走，反而还故意向后拽她。 |
| 1. 他另外有了女人，生意也不好做，每天都来拿借款，真想死掉一了百了。 |
| 1. 某一天整修铺面的工人无意中发现，原来在完工的花岗岩下方竟有前人铺设的石块路面。 |
| 1. 一股为牟利而破坏环境的歪风正迅速的蔓延开来。 |
| 1. 家长被迫教导子弟，要随时防范别人，极端的甚至强调即使面对熟人也不得不防。 |
| 1. 在今日的世界里，屋顶充斥着发射机和电视天线，传送或接收世界各个角落的各种讯息。 |
| 1. 今年暑假的玩具市场让恐龙出尽了风头，各大玩具店与礼品店，几乎都可见到恐龙玩具。 |
| 1. 漫步乡间小路，总有和善的笑脸自然相迎，走过古老农宅，又听到亲切的招呼殷勤相唤。 |
| 1. 香港一般的报纸，对政府干预市场对付炒家都表达了有限度支持的立场。 |
| 1. 经过重新改装的赛车，马力可以超过120匹，最快能够达到250公里以上的时速。 |
| 1. 俗称淮山的山药，基隆市每年产量都不少，但一般家庭只把它用作烹调。 |
| 1. 世界各国的菜肴中，不乏使用柠檬汁，但像巴西菜这么频繁的用法，还真是少见。 |
| 1. 他们很感兴趣，想先采购一批试试看销路怎么样，数量是一万支。 |
