# Supplementary material for: Effects of Spacing on Sentence Reading in Chinese
Source: Front Psychol. 2021 Nov 10;12:765335. doi: 10.3389/fpsyg.2021.765335 (PMC8631542; doi:10.3389/fpsyg.2021.765335)
Supplement: Supplementary file 2 [file Data_Sheet_2.docx]

**Supplementary materials S1.** Linear mixed-effects models

**Experiment 1**

***Word level measures***

*First Fixation Duration*

Note: The reference level of Spacing is unspaced. Trial number is the sentence position in the experiment. Length stands for word length in characters, and Word position controls for positioning of a word in a sentence.

|  | Estimate | SE | t-value | p-value |
| --- | --- | --- | --- | --- |
| Intercept | 5.332 | 0.018 | 289.745 | <0.001 |
| Spacing | -0.058 | 0.003 | -16.772 | <0.001 |
| Trial number | 0.003 | 0.001 | 1.871 | 0.061 |
| Length | 0.024 | 0.004 | 5.840 | <0.001 |
| Word position | 0.060 | 0.005 | 11.265 | <0.001 |

Table S1a: Fixed effects of the linear mixed-effects model fitted to first fixation duration.

| Random effect | Standard Deviation |
| --- | --- |
| Word | 0.037 |
| Sentence number | 0.023 |
| Participant | 0.310 |

Table S1b. Random effects of the linear mixed-effects model fitted to first fixation duration, including random intercepts for word, sentence number and participant. The R^2^ of the model is 0.12 and the standard deviation of the residual is 0.31. N before trimming 59154, N after trimming = 57720.

*Gaze Duration*

Note: The reference level of Spacing is unspaced. Trial number is the sentence position in the experiment. Length stands for word length in characters, and Word position controls for positioning of a word in a sentence.

|  | Estimate | SE | t-value | p-value |
| --- | --- | --- | --- | --- |
| Intercept | 5.353 | 0.020 | 2655.319 | <0.001 |
| Spacing | -0.066 | 0.004 | -16.778 | <0.001 |
| Trial number | -0.003 | 0.002 | -1.714 | 0.087 |
| Length | 0.053 | 0.005 | 11.361 | <0.001 |
| Word position | 0.031 | 0.006 | 5.001 | <0.001 |

Table S2a: Fixed effects of the linear mixed-effects model fitted to gaze duration.

| Random effect | Standard Deviation |
| --- | --- |
| Word | 0.054 |
| Sentence number | 0.028 |
| Participant | 0.111 |

Table S2b. Random effects of the linear mixed-effects model fitted to gaze duration, including random intercepts for word, sentence number and participant. The R^2^ of the model is 0.12 and the standard deviation of the residual is 0.35. N before trimming 59154, N after trimming = 57751.

*Total reading time on a word*

Note: The reference level of Spacing is unspaced. Trial number is the sentence position in the experiment. Length stands for word length in characters, and Word position controls for positioning of a word in a sentence.

|  | Estimate | SE | t-value | p-value |
| --- | --- | --- | --- | --- |
| Intercept | 5.575 | 0.025 | 224.342 | <0.001 |
| Spacing | -0.097 | 0.005 | -18.450 | <0.001 |
| Trial number | -0.023 | 0.002 | -10.689 | <0.001 |
| Length | 0.083 | 0.006 | 12.927 | <0.001 |
| Word position | -0.074 | 0.008 | -8.808 | <0.001 |

Table S3a: Fixed effects of the linear mixed-effects model fitted to total reading time.

| Random effect | Standard Deviation |
| --- | --- |
| Word | 0.104 |
| Sentence number | 0.070 |
| Participant | 0.132 |

Table S3b. Random effects of the linear mixed-effects model fitted to total reading time, including random intercepts for word, sentence number and participant. The R^2^ of the model is 0.15 and the standard deviation of the residual is 0.45. N before trimming 59154, N after trimming = 58481.

***Sentence reading times***

Note: Length stands for sentence length in characters, the reference level of Spacing is unspaced, Trial number is the sentence position in the experiment.

|  | Estimate | SE | t-value | p-value |
| --- | --- | --- | --- | --- |
| Intercept | 8.084 | 0.048 | 168.324 | <0.001 |
| Length (scaled) | 0.084 | 0.011 | 7.564 | <0.001 |
| Spacing | 0.007 | 0.008 | 0.864 | 0.388 |
| Trial number | -0.001 | 0.000 | -17.544 | <0.001 |

Table S4a: Fixed effects of the linear mixed-effects model fitted to total sentence reading times.

| Random effect | Standard Deviation |
| --- | --- |
| Sentence number | 0.154 |
| Participant | 0.268 |

Table S4b. Random effects of the linear mixed-effects model fitted to total sentence reading times, including random intercepts for sentence number and participant. The R^2^ of the model is 0.51 and the standard deviation of the residual is 0.32. N before trimming 7036, N after trimming = 6944. BIC for the model is 4597.485.

|  | Estimate | SE | t-value | p-value |
| --- | --- | --- | --- | --- |
| Intercept | 8.088 | 0.048 | 168.962 | <0.001 |
| Length (scaled) | 0.084 | 0.011 | 7.565 | <0.001 |
| Trial number | -0.001 | 0.000 | -17.546 | <0.001 |

Table S5a: Fixed effects of the linear mixed-effects model fitted to total sentence reading times without Spacing.

| Random effect | Standard Deviation |
| --- | --- |
| Sentence number | 0.153 |
| Participant | 0.268 |

Table S5b. Random effects of the linear mixed-effects model fitted to total sentence reading times without Spacing as a fixed effect, including random intercepts for sentence number and participant. The R^2^ of the model is 0.51 and the standard deviation of the residual is 0.32. N before trimming 7036, N after trimming = 6944. BIC for the model is 4581.49.

Bayes Factor was calculated as follows:

exp(0.5*(4581.49 – 4597.485)) = 0.00034

***Total saccade duration per sentence***

Note: The reference level of Spacing is spaced. Trial number is the sentence position in the experiment. Length stands for sentence length in characters.

|  | Estimate | SE | t-value | p-value |
| --- | --- | --- | --- | --- |
| Intercept | 5.574 | 0.054 | 120.630 | <0.001 |
| Spacing | 0.077 | 0.017 | 4.568 | <0.001 |
| Trial number | -0.001 | 0.000 | -14.663 | <0.001 |
| Length | 0.088 | 0.008 | 10.409 | <0.001 |

Table S6a: Fixed effects of the linear mixed-effects model fitted to total saccade duration.

| Random effect | Standard Deviation |
| --- | --- |
| Sentence number | 0.145 |
| Participant | 0.306 |

Table S6b. Random effects of the linear mixed-effects model fitted to total saccade duration, including random intercepts for sentence number and participant. The R^2^ of the model is 0.50 and the standard deviation of the residual is 0.36. N before trimming 6802, N after trimming = 6673.

***Total number of saccades per sentence***

Note: The reference level of Spacing is spaced. Length stands for sentence length in characters.

|  | Estimate | SE | t-value | p-value |
| --- | --- | --- | --- | --- |
| Intercept | 16.855 | 0.720 | 23.414 | <0.001 |
| Spacing | 1.016 | 0.280 | 3.631 | <0.001 |
| Length | 0.985 | 0.166 | 5.935 | <0.001 |

Table S7a: Fixed effects of the linear mixed-effects model fitted to total number of saccades.

| Random effect | Standard Deviation |
| --- | --- |
| Sentence number | 2.535 |
| Participant | 4.036 |

Table S7b. Random effects of the linear mixed-effects model fitted to total number of saccades, including random intercepts for sentence number and participant. The R^2^ of the model is 0.50 and the standard deviation of the residual is 4.94. N before trimming 6636, N after trimming = 6463.

**Experiment 2**

**Word level analysis**

*Gaze Duration*

Note: The reference level of Spacing is unspaced. Trial number is the sentence position in the experiment. Length stands for word length in characters, and Word position controls for positioning of a word in a sentence.

|  | Estimate | SE | t-value | p-value |
| --- | --- | --- | --- | --- |
| Intercept | 5.263 | 0.026 | 204.174 | <0.001 |
| Spacing | -0.010 | 0.005 | -2.056 | 0.041 |
| Trial number | -0.006 | 0.003 | -1.847 | 0.065 |
| Length | 0.114 | 0.002 | 48.628 | <0.001 |
| Word position | -0.015 | 0.011 | -1.317 | 0.188 |

Table S8a: Fixed effects of the linear mixed-effects model fitted to gaze duration.

| Random effect | Standard Deviation |
| --- | --- |
| Word | 0.107 |
| Sentence number | 0.028 |
| Participant | 0.145 |

Table S8b. Random effects of the linear mixed-effects model fitted to gaze fixation duration, including random intercepts for word, sentence number and participant. The R^2^ of the model is 0.27 and the standard deviation of the residual is 0.42. N before trimming 46386, N after trimming = 45482.

***Sentence reading times***

Note: Length stands for sentence length in characters, the reference level of Spacing is unspaced. Trial number is the sentence position in the experiment.

|  | Estimate | SE | t-value | p-value |
| --- | --- | --- | --- | --- |
| Intercept | 8.173 | 0.052 | 158.179 | <0.001 |
| Length (scaled) | 0.102 | 0.011 | 8.905 | <0.001 |
| Spacing | -0.022 | 0.007 | -3.317 | <0.001 |
| Trial number | -0.001 | 0.000 | -5.050 | <0.001 |

Table S9a: Fixed effects of the linear mixed-effects model fitted to total sentence reading times.

| Random effect | Standard Deviation |
| --- | --- |
| Sentence number | 0.136 |
| Participant | 0.287 |

Table S9b. Random effects of the linear mixed-effects model fitted to total sentence reading times, including random intercepts for sentence number and participant. The R^2^ (conditional) of the model is 0.55 and the standard deviation of the residual is 0.30. N before trimming 7987, N after trimming = 7876.
